# The Use of Problem-Solving Therapy for Primary Care to Enhance Complex Decision-Making in Healthy Community-Dwelling Older Adults

**DOI:** 10.3389/fpsyg.2018.00870

**Published:** 2018-06-13

**Authors:** Christopher M. Nguyen, Kuan-Hua Chen, Natalie L. Denburg

**Affiliations:** ^1^Division of Cognitive Neuroscience, Department of Neurology, University of Iowa Carver College of Medicine, Iowa City, IA, United States; ^2^Department of Psychiatry and Behavioral Sciences, University of Oklahoma Health Sciences Center, Oklahoma City, OK, United States; ^3^Institute of Personality and Social Research, University of California, Berkeley, Berkeley, CA, United States

**Keywords:** aging, decision making, executive functioning, Iowa Gambling Task, Problem-Solving Therapy for Primary Care

## Abstract

Some older adults who are cognitively healthy have been found to make poor decisions. The vulnerability of such older adults has been postulated to be the result of disproportionate aging of the frontal lobes that contributes to a decline in executive functioning abilities among some older adults. The purpose of this study was to investigate whether decision-making performance in older adults can be enhanced by a psychoeducational intervention. Twenty cognitively and emotionally intact persons aged 65 years and older were recruited and randomized into two conditions: psychoeducational condition [Problem-Solving Therapy for Primary Care (PST-PC)] and no-treatment Control group. Participants in the psychoeducational condition each received four 45-min sessions of PST-PC across a 2-week period. The Iowa Gambling Task (IGT) was administered as the outcome measure to the treatment group, while participants in the Control group completed the IGT without intervention. A significant interaction effect was observed between group status and the trajectory of score differences across trials on the IGT. Particularly, as the task progressed to the last 20% of trials, participants in the PST-PC group significantly outperformed participants in the Control group in terms of making more advantageous decisions. These findings demonstrated that a four-session problem-solving therapy can reinforce aspects of executive functioning (that may have declined as a part of healthy aging), thereby enhancing complex decision-making in healthy older adults.

## Introduction

The ability of older adults to make sound decisions regarding retirement, allocation of resources, living arrangements, health insurance, and medical procedures has a profound effect on the well-being of the individual as well as society, cumulatively. Even older adults who are cognitively healthy, without a neurodegenerative disease or mild cognitive impairment, have been found to make poor decisions (Denburg et al., 2007). Specifically, some older adults fail to make advantageous decisions and become susceptible to scams, make poor financial decisions, or experience abuse of trust and get taken advantage of by others. These forms of financial exploitation have been reported to increase dramatically among older adults (Lichtenberg et al., 2015).

The weaknesses in decision-making capacity among older adults have been postulated to be triggered by a distinct neurological change: disproportionate age-related decline of the frontal lobes of the brain (West, 1996). In particular, the frontal lobe hypothesis of cognitive aging posits that cognitive abilities dependent on the frontal regions of the brain would experience a disproportionate age-related decline, whereas other functioning independent of the frontal lobes will remain relatively intact (West, 1996). This theory has gained support from multiple neuroscience disciplines, including neuropsychology, neuroanatomy, and functional neuroimaging (see review by, Reuter-Lorenz et al., 2016). The reasons why some older adults are vulnerable and susceptible to making poor decisions have been examined thoroughly through neurobiological and behavioral mechanisms, and research on applied contexts has been important to understanding day-to-day decisions (Hess et al., 2015). Much of the current research on aging and decision making in applied domains has focused on the implication of decisions in various contexts such as medical decision-making (Leventhal et al., 2015), health-related decisions (Liu et al., 2015), and consumer decision-making (Carpenter and Yoon, 2015). Yet, research efforts examining interventions to enhance older adults decision-making abilities are lacking.

Deficits in decision-making may be a function of weaknesses in executive functioning. Specifically, executive functions involve abilities such as planning, organization, goal setting, initiation, and utilization of feedback and attention shifting – all essential skills necessary in the process of decision-making. A review of the treatment modalities revealed that Problem-Solving Therapy for Primary Care (PST-PC; Hegel and Arean, 2003) is one treatment modality to have demonstrated efficacy in managing such executive dysfunction (Alexopoulous et al., 2003).

Problem-Solving Therapy for Primary Care was developed as an efficient modality to treat patients in busy primary care settings over the course of 4–8 sessions. It has been found that as few as three sessions of PST-PC could be beneficial (Mynors-Wallis et al., 2000; Hegel et al., 2004; Arean et al., 2008). Trained specialists can deliver PST-PC after undergoing a brief training module (Hegel et al., 2004; Arean et al., 2008). Furthermore, PST-PC has been demonstrated to be as effective when implemented by nurses and primary care physicians as compared to implementation by mental health professionals (Mynors-Wallis et al., 2000; Unutzer et al., 2002). When comparing PST-PC with antidepressants among depressed patients, PST-PC has been shown to be just as efficacious in improving psychological symptoms and social functioning (Mynors-Wallis et al., 1995). Furthermore, the effectiveness of PST-PC has been evaluated in several randomized-controlled trials to treat various psychological problems including depression, anxiety, and insomnia (Dowrick et al., 2000; Mynors-Wallis et al., 2000).

Problem-Solving Therapy for Primary Care has been demonstrated as an efficacious intervention to improve mood and cognitive functioning in elderly depressed patients (Alexopoulous et al., 2003; Arean et al., 2008, 2010). When compared with other treatment modalities such as cognitive-behavioral therapy and psychodynamic approaches, depressed older adults who were treated with PST-PC reported fewer depressive symptoms and improved functioning at 12 months and up to 24 months follow-up (Arean et al., 2008). Elderly depressed patients receiving PST-PC treatments have exhibited reduction of symptoms, endorsed higher response rate to treatment, and greater remission rate when compared with those receiving a person-centered psychotherapy treatment approach (Arean et al., 2010). Among depressed elderly patients with impairments in aspects of executive functioning, those receiving PST-PC treatments (versus supportive counseling) demonstrated greater improvement in generating alternative solutions and decision-making skills, in addition to reduced depressive symptoms and improved functioning (Alexopoulous et al., 2003).

The purpose of this study was to investigate whether decision-making performance among healthy community-dwelling older adults can be improved by a brief four-session (approximately 2 weeks) problem-solving therapy modality. To our knowledge, this is one of the first studies to introduce a psychosocial intervention to enhance complex decision-making among healthy community-dwelling older adults in an outpatient context. PST-PC was specifically chosen due to its: 1) efficacy among older adults; 2) efficient protocol that can be delivered in four sessions (or in our case, about 2 weeks); and 3) effectiveness when implemented by trained individuals not in the field of mental health (Arean, 2009). Morever, the cognitive changes associated with aging have demonstrated age-related effects in prefrontal brain structures contributing to weaknesses in aspects of executive functioning (e.g., planning, initiation, decision-making, and problem-solving), and thus the utilization of PST-PC as a possible compensatory strategy to address these deficits can be a valuable form of intervention. It was hypothesized that decision-making performance among healthy community-dwelling older adults would improve for those in the PST-PC condition when compared to participants in the no-treatment Control condition.

## Materials and Methods

### Participants

Participants were included in the study if they were heathy, community-dwelling, aged 65 years and older, and cognitively and emotionally intact, and were excluded from the study if they had any major underlying medical conditions (e.g., cancer, cardiovascular disease, and movement disorder). Participants were recruited from a pool of participants involved in an ongoing project investigating the neural correlates of decision-making. These participants were evaluated extensively, with both clinical interview and comprehensive neuropsychological assessment, and thus were deemed cognitively and emotionally intact (after Tranel et al., 1997). Participants completed an informed consent process approved by an Institutional Review Board, and were financially compensated for their involvement. Twenty participants were randomly assigned to two groups: psychoeducational condition (PST-PC) and no-treatment Control group. The PST-PC group (*n* = 10) had a mean age of 80.5 years [standard deviation (SD) = 3.5] and 50% males. The Control group (*n* = 10) had a mean age of 80.0 years (*SD* = 4.3) and 50% males.

### Procedures

All participants completed a 2-h comprehensive battery of neuropsychological tests to assess a broad range of cognitive abilities. A research assistant with training in neuropsychological assessment administered the test battery under the supervision of a neuropsychologist (NLD). Ten domains were assessed with the following administered instruments: general mental status (Folstein Mini-Mental State Examination; Folstein et al., 1975); estimated premorbid IQ (Wide Range Achievement Test-3; Wilkinson, 1993); verbal and non-verbal intellectual functioning (Wechsler Abbreviated Scale of Intelligence; Wechsler, 1999); attention and working memory (Wechsler Adult Intelligence Scale-III Working Memory Index; Wechsler, 1997); processing speed (Trail Making Test Part A; Spreen and Strauss, 1998); language (Controlled Oral Word Association Test; Benton and Hamsher, 1989); learning and memory [Rey Auditory Verbal Learning Test (Rey, 1941) and Rey–Osterrieth Complex Figure Test (Rey, 1964)]; visuoperception (Benton Facial Discrimination Test; Benton et al., 1994); mental flexibility and set-shifting (Trail Making Test Part B; Spreen and Strauss, 1998); and mood (Beck Depression Inventory-II; Beck et al., 1996).

Participants randomized into the psychoeducational condition each completed four 45-min sessions of the PST-PC protocol during a 2-week period. A doctoral candidate in psychology (CMN) with training in cognitive-behavioral therapy delivered the PST-PC sessions following a manualized protocol to all participants under the supervision of a licensed clinical psychologist (NLD). Through a seven-step model of PST-PC (Hegel and Arean, 2003), participants identified problems to be solved; discussed and evaluated different resolutions to reach desired goals; created action plans to accomplish determined goals; and evaluated their effectiveness in resolving designated problems. In the first session, the structure of the treatment process was outlined, and the seven stages of the problem-solving process were thoroughly discussed. In the second session, the participant selected one problem from the list generated in the first session to be resolved. The seven stages of PST-PC were integrated during the process of identifying the problem to be resolved and formation of the action plan. During the third session, participants evaluated the outcomes of their action plans. This session consisted of a discussion on how well they have integrated the seven stages of PST-PC toward the resolution of a designated problem to be resolved. If a participant successfully resolved the problem, a new problem was selected, and the process was discussed in the last session. If a participant was not successful in resolving a designated problem, the next session was used to further discuss progress or setbacks. The final session was used to review the participants’ progress and reinforce continued efforts in resolution of future problems (Hegel and Arean, 2003).

All but one participant completed the PST-PC protocol at our clinic. For this one individual, the sessions were completed at their home due to limited mobility secondary to a recent orthopedic surgery. There was a 3- to 4-day interval between each of the four sessions. Participants were scheduled to complete the outcome measure within 3 days of completing the final PST-PC session. The participants from the Control group were recruited and scheduled to complete the outcome measure.

### Manipulation Check

A manipulation check was applied to confirm that the seven stages of the PST-PC protocol was successfully implemented. At baseline and post-PST-PC sessions, participants were asked to respond to one open-ended essay-format question, as follows: “Please describe the process of problem-solving in detail, including all steps and the criteria for successfully completing each one.” All essays were scored using criteria designated in the 20-item Problem-Solving Treatment Knowledge Assessment (PST-KA; Cartreine et al., 2012), based on how well each essay discussed the stages of Problem-Solving Treatment (e.g., Identifying the Problem, Setting a Goal, Brainstorming Solutions, Selecting Solutions for Implementation, and Action Planning). Each item on the PST-KA was rated on a six-point scale (0–5; very poor to very good). The possible range of scores was 0–100, with higher scores indicating greater baseline knowledge/knowledge obtained (hereafter refered to as closure knowledge). Two research assistants who were blind to the time point of the completed essays completed the ratings. An average score was calculated across scores from the research assistants.

### Decision-Making Outcome Measure

The Iowa Gambling Task (IGT; Bechara, 2007) is a measure of complex decision-making under ambiguity that features real-world aspects of reward, punishment, and unpredictability. The IGT is a computer-administered test comprised of 100 card selections from four decks of cards. On each trial, choosing a card gives an immediate monetary reward. At random points, the selection of some cards results in losing a sum of money. Two decks of cards are predetermined to offer a lower immediate gain and even lower long-term loss, yielding an overall net gain of money (i.e., referred to as “the good decks”). Alternatively, the other two decks are predetermined to offer a higher immediate gain but even higher long-term loss, yielding an overall net loss of money (i.e., referred to as “the bad decks”). Participants are not informed of the number of trials or the gain/loss patterns.

Performance on the IGT is often quantified by dividing the 100 trials into five distinct blocks of 20 trials each to examine participant’s learning curve (Bechara, 2007). A score for each block is calculated by subtracting the number of selection from the good decks from the number of selections from the bad decks, while a total score for the IGT is calculated by subtracting the total number of selections from the bad decks from the total number of selections from the good deck. A positive total score indicates advantageous decision-making, whereas a negative total score indicates disadvantageous decision-making (Bechara, 2007).

### Statistical Analysis

Preliminary analysis examined the data for the presence of outliers. Independent samples *t*-tests were employed to examine differences between the participant groups on demographic variables, cognitive performance, and mood. Next, a paired-samples *t*-test was conducted to examine whether a mean difference existed in baseline and/or closure knowledge after four sessions of PST-PC. Finally, to explore the effects of PST-PC on the decision-making outcome measure, a 2x5 repeated measures analysis of variance (ANOVA) using group status (PST-PC versus Control) as the between-subjects factor and trial block (1–5) as the within-subjects factor was employed to evaluate performance on the IGT outcome measure by trial block.

## Results

**FIGURE 1:**
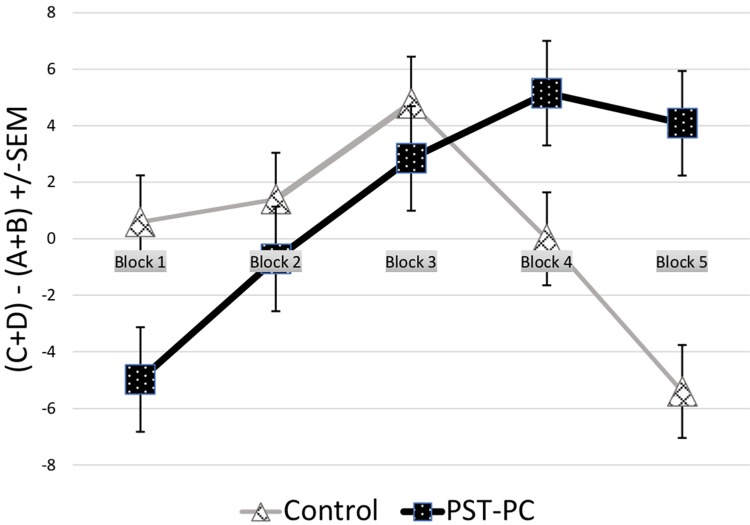
Iowa Gambling Task scores by trail block for PST-PC and Control groups. Decision-making performance on the IGT in PST-PC and Control participants, graphed as a function of trial block [±SEM (standard error of the mean)]. A significant interaction effect was indicated between group status and the trajectory of score differences across the five trial blocks on the IGT, revealing a statistically significant difference between the two groups in advantageous versus disadvantageous selections on the last block of the IGT, or during the last 20% of selections.

The two participant groups did not significantly differ in terms of education, general mental status, estimated premorbid IQ, verbal and non-verbal intellectual functioning, attention and working memory, processing speed, language, learning and memory, visuoperception, mental flexibility and set-shifting, and mood. Demographic and cognitive characteristics are presented in **Table 1**. A paired-samples *t*-test comparing pre- and post-intervention PST-KA scores of participants from the PST-PC group revealed a significant difference in the baseline knowledge assessment scores (*M* = 22.0; *SD* = 13.3) as compared to the closure knowledge scores (*M* = 38.8, *SD* = 19.6); *t*(9) = -2.3, *p* = 0.047. This implied that the seven stages of the PST-PC protocol was successfully implemented. A repeated measures ANOVA revealed a non-significant main effect for group on IGT scores, *F*(4,15) = 2.04, *p* = 0.097. Although there were no significant group differences with the overall IGT index, descriptive statistics revealed that four (of 10) participants in the Control group achieved an overall index in IGT scores that were below zero (range: -16 to -44), as compared to none from the PST group. Of note, IGT scores that were significantly below zero have been classified as an “Impaired” performance in past studies (Denburg et al., 2005). A significant interaction effect was indicated between group status and the trajectory of score differences across the five trial blocks on the IGT, *F*(4,15) = 3.24, *p* = 0.017, which is indicative that group status had different effects on participant’s learning curve on the IGT trials, as presented in **Figure 1**. To explore this interaction, contrasts were performed for individual trial blocks, revealing a statistically significant difference between the two groups in advantages versus disadvantageous selections on the last block of the IGT, *t*(18) = -3.02, *p* = 0.007, *d* = 1.35, 95% CI [-22, -4]. Particularly, as the task progressed to the end, participants in the PST-PC group significantly outperformed participants in the Control group in terms of making more advantageous decisions in the last 20% of trials (or card selections 81–100).

**Table 1:** Demographic and cognitive characteristics.

Characteristics	Participant group			
	PST (*n* = 10) *M* (*SD*)	Control (*n* = 10) *M* (*SD*)	*t*(18)	*P*-value
Age	80.5 (3.5)	80.0 (4.3)	-0.28	0.782
Sex (% female)	50%	50%	–	–
Handedness (% right)	100%	80%	–	–
Education (years)	14.9 (2.2)	16.2 (3.3)	1.03	0.319
MMSE	29.2 (0.8)	28.8 (1.1)	-0.92	0.372
WRAT-3 reading	106.2 (7.8)	110.0 (6.8)	1.16	0.262
WASI VCI	119.1 (9.6)	117.2 (11.2)	-0.39	0.705
WASI PRI	114.5 (12.5)	118.6 (14.6)	0.65	0.522
WAIS-III WMI	109.8 (10.9)	117.1 (15.8)	1.13	0.277
Trail Making Test-A (s)	36.5 (13.9)	29.2 (6.9)	-1.48	0.155
Trail Making Test-B (s)	81.0 (32.9)	64.4 (16.2)	-1.43	0.170
COWAT (raw)	45.3 (15.0)	46.3 (12.1)	0.16	0.871
Benton faces (raw)	47.8 (4.7)	46.6 (4.3)	-0.55	0.592
Rey-O copy	31.2 (3.0)	31.8 (4.1)	-0.88	0.390
RAVLT 1–5 total raw	48.0 (4.7)	51.3 (8.3)	1.10	0.288
RAVLT 30-min delay	10.3 (2.3)	9.7 (2.4)	-0.57	0.577
Rey-O 30-min delay	18.3 (8.1)	18.4 (7.2)	0.03	0.977
BDI-II (raw)	3.4 (3.2)	4.6 (3.4)	0.75	0.463

*Shown are Folstein Mini-Mental State Examination (MMSE); Wide Range Achievement Test-3 (WRAT-3) Reading subtest (in Standard Scores); Wechsler Abbreviated Scale of Intelligence (WASI) Verbal Comprehension Index (VCI) and Performance Reasoning Index (PRI) (in Standard Scores); Wechsler Adult Intelligence Scale-Third Edition (WAIS-III) Working Memory Index (WMI) (in Standard Scores); Controlled Oral Word Association Test (COWAT); Rey Auditory Verbal Learning Test (RAVLT), Rey–Osterrieth Complex Figure (Rey-O); and Beck Depression Inventory-II (BDI-II).*

## Discussion

The purpose of this study was to investigate whether decision-making performance among healthy community-dwelling older adults could be improved as a result of a well-validated psychoeducational intervention. Twenty participants were recruited and randomized into two conditions: PST-PC and a no-treatment Control group. The theoretical framework of this study is based on a body of literature suggesting that a disproportionate deterioration of the frontal lobes during aging contributes to a decline in executive functioning abilities among some older adults (West, 1996). Previous work from our laboratory have supported this “frontal lobe hypothesis,” revealing that seemingly healthy older adults often make disadvantageous decisions (Denburg et al., 2005, 2006, 2007). Specifically, we have found that some older adults may experience a greater decline in non-memory-related cognitive functioning, such as problem-solving and mental flexibility, contributing to weaknesses in their decision-making abilities (Denburg and Hedgcock, 2015). The findings from the current study demonstrated that a four-session (approximately 2 weeks) problem-solving therapy can reinforce aspects of executive functioning (that may have declined as a part of healthy aging), thereby enhancing decision-making abilities.

With regard to decision-making outcomes, the proportion of our participants with “impaired” and “unimpaired” performance on the IGT from the Control group is comparable to findings from previous studies using this classification. Specifically, Denburg et al. (2005, 2006) defined “impaired” performance on the IGT as being significantly worse than performance at chance level, and found that approximately 25–35% of their older adult sample performed in the “impaired” range. Additionally, this finding is consistent with another study suggesting that a subset of older adults make less advantageous decisions when compared to younger adults (Fein et al., 2007).

Interestingly, earlier findings by Bechara et al. (1997) have indicated that by the 80th card selection (out of 100), normal healthy young adults would reach a “conceptual period” during which they exhibited knowledge regarding optimal choices based on prior feedback and typically avoid the disadvantage selections. Notably, as the task progressed to the latter 20% of the task, participants in the PST-PC group significantly outperformed participants in the Control group in terms of making more advantageous decisions. Group differences emerged as the IGT progressed such that those in the PST-PC group learned to adapt to feedback that led to making more advantageous decisions. Alternatively, participants without the benefit of the PST-PC psychoeducation treatment (i.e., the Control group) shifted between decks and were inefficient in developing a strategy over time which may have contributed to the overall less advantageous choices than participants in the PST-PC group.

It has been postulated that individuals exhibiting difficulty in developing an advantageous and stable strategy over time on the IGT is likely to be related to weaknesses in aspects of executive functioning (Okdie et al., 2016). Furthermore, an inflexibility in responding to negative feedback after a disadvantageous decision has been postulated to be related to poor executive functioning, which results in an individual being less likely to adapt to the feedback to choose more advantageous options (Zamarian et al., 2008). The findings from this study suggests that PST-PC may be effective in generating an efficient learning process that contributes to advantageous decision-making outcomes.

The components taught during the various PST-PC sessions provide an opportunity for the individual to broadly strengthen executive skills referenced by Lezak et al. (2012), such as emotional regulation, behavioral initiation, planning, organization, cognitive flexibility, and problem-solving. A person with weaknesses in executive dysfunction can be overwhelmed with complex tasks and situations, which can be remediated during the initial stages of PST-PC through a structured approach to problem solving (e.g., breaking down complex problems into small and manageable parts). Cognitive flexibility is facilitated during the brainstorming stages of PST-PC, where individuals are encouraged to generate multiple solutions toward a satisfactory resolution of a problem. Aspects of executive functioning such as planning and organization are facilitated during the middle phases of PST-PC, where individuals evaluate and compare solutions generated during the brainstorming step to determine the best selection to be implemented. Behavioral initiation is fostered through the development of an action plan during the later steps of PST-PC. Overall, the process of implementing the stages of PST-PC requires abstract problem-solving with inductive reasoning and flexible adjustment of responses based on feedback, and may have contributed to improved decision-making outcomes.

A psychoeducational approach such as PST-PC can contribute to increased self-efficacy among older adults and improve decision-making abilities. To illustrate, it has been suggested that interventions can be more efficacious when integrating older adults’ strengths, such as their life experiences, to increase self-efficacy (e.g., positivity, confidence, and motivation) (Strough et al., 2015). Furthermore, to improve their sense of self-efficacy, individuals must be engaged in an activation process that facilitates the examination of his/her knowledge, skills, and confidence with respect to the relevant topic requiring a decision to be made, and then formulating a concrete action plan to be implemented (Hibbard and Mahoney, 2010). The latter stages of PST-PC evoked this process, when participants were asked to evaluate and compare solutions generated through brainstorming, and to determine the best selection to be implemented as an action plan. Furthermore, solicited feedback from the participants revealed that the psychoeducational component of PST-PC provided during the initial sessions solidified and enhanced preexisting knowledge and approaches to problem-solving (i.e., promoting self-efficacy from life experiences), in addition to providing a structured approach to facilitate a more efficient process for resolving practical everyday challenges.

This is one of the first studies to adapt PST-PC for use as an intervention to enhance decision-making in healthy community-dwelling older adults. By contrast, much of the extant literature in facilitation of advantageous decision-making outcomes relies extensively on the use of decision aids, or interventions designed to assist in the deliberation between treatment options by provided content-related information (e.g., health-related information when choosing between medical treatment options) (Stacey et al., 2014). While these decision aids have been found to be effective in increasing knowledge and risk perception as well as contributing to a more well-informed decision-making process, few studies have explicitly examined its effectiveness among older adults (van Weert et al., 2016). Incidentally, a majority of participants in this study readily identified a common health-related theme (e.g., weight loss, managing high cholesterol, improving sleep hygiene, and managing chronic pain) when asked to identify a problem to be applied during the PST-PC protocol. Perhaps this is suggestive that PST-PC can be utilize as a modality to facilitate more active participation (as compared to decision aids) among older adults in enhancing aspects of complex decision-making processes in the healthcare arena.

This study is not without its limitations. Participants in our study were highly educated (e.g., 70% with 16 years of education and above) for an older adult sample and performed in the high average range on measures of general intellectual functioning. By contrast, the 2015 Census data reported that only 27% of the population 65 years of age and older had earned a bachelor’s degree or more (He et al., 2005). Finally, the present study had a relatively small sample size and was homogenous in terms of race (i.e., all participants were non-Hispanic, white). These issues may limit the generalizability of our findings. Another limitation of the study is the utilization of a single laboratory measure of decision making. While the IGT has been a well-validated measure to detect decision-making deficits (Bechara, 2007), decision-making is complex and multifaceted, and undoubtedly difficult to measure fully with any laboratory task. Future studies should validate the efficacy of PST-PC in enhancing decision-making outcomes among older adults in other applied tasks such as the Multiple Errands test (Tranel et al., 2007) or the Financial Decision-Making test (Shivapour et al., 2012).

## Ethics Statement

This study was carried out in accordance with the approval of University of Iowa Institutional Review Board with written informed consent from all subjects.

## Author Contributions

CMN and NLD: study concept, design, analysis, data interpretation, manuscript writing, data verification, and analysis. K-HC: study design.

## Conflict of Interest Statement

The authors declare that the research was conducted in the absence of any commercial or financial relationships that could be construed as a potential conflict of interest. The reviewer GD and handling Editor declared their shared affiliation.

## References

[B1] Alexopoulous G. S. Raue P. Arean P. (2003). Problem-solving therapy versus supportive therapy in geriatric major depression with executive dysfunction. *Am. J. Geriat. Psychiatry* 11 46–52. 10.1097/00019442-200301000-00007 12527539

[B2] Arean P. Hegel M. Vannoy S. Fan M. Y. Unuzter J. (2008). Effectiveness of problem-solving therapy for older, primary care patients with depression: results from the IMPACT project. *Gerontologist* 48 311–323. 10.1093/geront/48.3.311 18591356

[B3] Arean P. A. (2009). Problem-solving therapy. *Psychiatr. Ann.* 39 854–862. 10.3928/00485713-20090821-01

[B4] Arean P. A. Raue P. Mackin R. S. Kanellopoulous D. McCulloch C. Alexopoulous G. S. (2010). Problem-solving therapy and supportive treatment in older adults with major depression and executive dysfunction. *Am. J. Psychiatry* 167 1391–1398. 10.1001/archgenpsychiatry.2010.177 20516155 PMC2998516

[B5] Bechara A. (2007). *Iowa Gambling Task (IGT) Professional Manual.* Lutz: Psychological Assessment Resources.

[B6] Bechara A. Damasio H. Tranel D. Damasio A. R. (1997). Deciding advantageously before knowing the advantageous strategy. *Science* 275 1293–1295. 10.1126/science.275.5304.1293 9036851

[B7] Beck A. T. Steer R. A. Brown G. (1996). *Beck Depression Inventory II: Manual.* San Antonio, TX: Psychological Corporation.

[B8] Benton A. L. Hamsher K. (1989). *Multilingual Aphasia Examination.* Iowa, IA: AJA Associates.

[B9] Benton A. L. Sivan A. B. Hamsher K. Varney N. R. Spreen O. (1994). *Contributions to Neuropsychological Assessment: A Clinical Manual*, 2nd Edn. New York, NY: Oxford University Press.

[B10] Carpenter S. M. Yoon C. (2015). “Aging and consumer decision making,” in *Aging and Decision Making: Empirical and Applied Perspectives*, eds Hess T. M. Strough J. Lockenhoff C. E. (New York, NY: Academic Press), 351–371.

[B11] Cartreine J. A. Chang T. E. Seville J. L. Sandoval L. Moore J. B. Xu S. (2012). Using self-guided treatment software (ePST) to teach clinicians how to deliver problem-solving treatment for depression. *Depress. Res. Treat.* 2012:309094. 10.1155/2012/309094 23213493 PMC3505632

[B12] Denburg N. L. Hedgcock W. M. (2015). “Age-associated executive dysfunction, the prefrontal cortex, and complex decision making,” in *Aging and Decision Making: Empirical and Applied Perspectives*, eds Hess T. M. Strough J. Lockenhoff C. E. (New York, NY: Academic Press), 81–104.

[B13] Denburg N. L. Cole C. A. Hernandez M. Yamada T. H. Tranel D. Bechara A. (2007). The orbitofrontal cortex, real-world decision making, and normal aging. *Ann. N. Y. Acad. Sci.* 1121 480–498. 10.1196/annals.1401.031 17872394 PMC2246008

[B14] Denburg N. L. Recknor E. C. Bechara A. Tranel D. (2006). Psychophysiological anticipation of positive outcomes promotes advantageous decision-making in normal older persons. *Int. J. Psychophysiol.* 61 19–25. 10.1016/j.ijpsycho.2005.10.021 16426691

[B15] Denburg N. L. Tranel D. Bechara A. (2005). The ability to decide advantageously declines prematurely in some normal older persons. *Neuropsychologia* 43 1099–1106. 10.1016/j.neuropsychologia.2004.09.012 15769495

[B16] Dowrick C. Dunn G. Ayuso-Mateos J. L. Dalgard O. S. Page H. Lehtinen V. (2000). Problem solving treatment and group psychoeducation for depression: multicentre randomized controlled trial. *Br. Med. J.* 321 1–6. 10.1136/bmj.321.7274.1450 11110739 PMC27549

[B17] Fein G. McGillivray S. Finn P. (2007). Older adults make less advantageous decisions than younger adults: cognitive and psychological correlates. *J. Int. Neuropsychol. Soc.* 13 480–489. 10.1017/S135561770707052X 17445297 PMC1868494

[B18] Folstein M. F. Folstein S. E. McHugh P. R. (1975). Mini-mental state”. A practical method for grading the cognitive state of patients for the clinician. *J. Psychiatry Res.* 12 189–198. 10.1016/0022-3956(75)90026-6 1202204

[B19] He W. Sengupta M. Velkoff V. A. DeBarros K. A. (2005). *65+ in the United States: 2005. U.S. Census Bureau, Current Population Reports.* Washington, DC: U.S. Government Printing Office, 23–209.

[B20] Hegel M. Arean P. A. (2003). *Problem-Solving Treatment for Primary Care: A Treatment Manual for Depression.* Lebanon, NH: Project IMPACT, Dartmouth College.

[B21] Hegel M. T. Dietrich A. J. Seville J. L. Jordan C. B. (2004). Training residents in problem-solving treatment of depression: a pilot feasibility and impact study. *Fam. Med.* 36 204–208. 14999578

[B22] Hess T. M. Strough J. Lockenhoff C. E. (2015). *Aging and Decision Making: Empirical and Applied Perspectives.* New York, NY: Academic Press.

[B23] Hibbard J. H. Mahoney E. R. (2010). Toward a theory of patient and consumer activation. *Patient Educ. Counsel.* 78 377–381. 10.1016/j.pec.2009.12.015 20188505

[B24] Leventhal H. Herold J. Leventhal E. A. Burns E. Diefenbach M. A. (2015). “Decisions and actions for life patterns and health practices as we age: a bottom-up approach,” in *Aging and Decision Making: Empirical and Applied Perspectives*, eds Hess T. M. Strough J. Lockenhoff C. E. (New York, NY: Academic Press), 283–308. 10.1016/B978-0-12-417148-0.00014-5

[B25] Lezak M. D. Howieson D. B. Bigler E. D. Tranel D. (2012). *Neuropsychological Assessment*, 5th Edn. New York, NY: Oxford University Press.

[B26] Lichtenberg P. A. Stoltman J. Ficker L. J. Iris M. Mast B. (2015). A person-centered approach to financial capacity: preliminary development of a new rating scale. *Clin. Gerontol.* 38 49–67. 10.1080/07317115.2014.970318 25866438 PMC4392714

[B27] Liu P. Wood S. Hanoch Y. (2015). “Choice and aging: less is more,” in *Aging and Decision Making: Empirical and Applied Perspectives*, eds Hess T. M. Strough J. Lockenhoff C. E. (New York, NY: Academic Press), 309–329. 10.1016/B978-0-12-417148-0.00015-7

[B28] Mynors-Wallis L. M. Gath D. H. Day A. Baker F. (2000). Randomized controlled trial of problem-solving treatment, antidepressant medication, and combined treatment for major depression in primary care. *Br. Med. J.* 320 26–30. 10.1136/bmj.320.7226.26 10617523 PMC27250

[B29] Mynors-Wallis L. M. Gath D. H. Lloyd-Thomas A. R. Tomlinson D. (1995). Randomised controlled trial comparing problem solving treatment with amitryptyline and placebo for major depression in primary care. *Br. Med. J.* 310 441–445. 10.1136/bmj.310.6977.441 7873952 PMC2548821

[B30] Okdie B. M. Buelow M. T. Bevelhymer-Rangel K. (2016). It’s all in how you think about it: construal level and the Iowa gambling task. *Front. Neurosci.* 10:2 10.3389/fnins.2016.00002 PMC4722111 26834531

[B31] Reuter-Lorenz P. A. Festini S. B. Jantz T. K. (2016). “Executive functions and neurocognitive aging,” in *Handbook of the Psychology of Aging*, 8th Edn, eds Schaie K. W. Willis S. (San Diego, CA: Elsevier), 245–262. 10.1016/B978-0-12-411469-2.00013-3

[B32] Rey A. (1941). L’examen psychologique dans les cas d’encephalopathie traumatique. *Arch. Psychol.* 28 215–285.

[B33] Rey A. (1964). *L’examen Clinique en Psychologie.* Paris: Presses Universitaires de France.

[B34] Shivapour S. K. Nguyen C. M. Cole C. A. Denburg N. L. (2012). Effects of age, sex, and neuropsychological performance on financial decision-making. *Front. Neurosci.* 6:82. 10.3389/fnins.2012.00082 22715322 PMC3375479

[B35] Spreen O. Strauss E. (1998). *A Compendium of Neuropsychological Tests*, 2nd Edn. New York, NY: Oxford University Press.

[B36] Stacey D. Légaré F. Col N. F. Bennett C. L. Barry M. J. Eden K. B. (2014). Decision aids for people facing health treatment or screening decisions. *Cochrane Database Syst. Rev.* 28:CD001431. 10.1002/14651858.CD001431.pub5 24470076

[B37] Strough J. de Bruine W. B. Peters E. (2015). New perspectives for motivating better decisions in older adults. *Front. Psychol.* 6:783. 10.3389/fpsyg.2015.00783 26157398 PMC4475788

[B38] Tranel D. Benton A. Olson K. (1997). A 10-year longitudinal study of cognitive changes in elderly persons. *Dev. Neuropsychol.* 13 87–96. 10.1080/87565649709540669

[B39] Tranel D. Hathaway-Nepple J. Anderson S. W. (2007). Impaired behavior on real-world tasks following damage to the ventromedial prefrontal cortex. *J. Clin. Exp. Neuropsychol.* 29 319–332. 10.1080/13803390600701376 17454352 PMC2289390

[B40] Unutzer J. Katon W. Callahan C. M. Williams J. W. Jr. Hunkeler E. Harpole L. (2002). Collaborative care management of late life depression in primary care: a randomized controlled trial. *J. Am. Med. Assoc.* 288 2836–2845. 10.1001/jama.288.22.2836 12472325

[B41] van Weert J. C. van Munster B. C. Sanders R. Spijker R. Hooft L. Jansen J. (2016). Decisions aids to help older people make health decisions: a systematic review and meta-analysis. *BMC Med. Inform. Decis. Mak.* 16:45. 10.1186/s12911-016-0281-8 27098100 PMC4839148

[B42] Wechsler D. (1997). *Wechsler Adult Intelligence Scale–III.* San Antonio, TX: Psychological Corp.

[B43] Wechsler D. (1999). *The WASI: Wechsler Abbreviated Scale of Intelligence.* San Antonio, TX: Psychological Corp.

[B44] West R. (1996). An application of prefrontal cortex function theory of cognitive aging. *Psychol. Bull.* 120 272–292. 10.1037/0033-2909.120.2.272 8831298

[B45] Wilkinson G. S. (1993). *WRAT-3: Wide Range Achievement Test Administration Manual*, 3rd Edn. Wilmington, DE: Wide Range.

[B46] Zamarian L. Sinz H. Bonatti E. Gamboz N. Delazer M. (2008). Normal aging affects decisions under ambiguity, but not decisions under risk. *Neuropsychology* 22 645–657. 10.1037/0894-4105.22.5.645 18763884

